# Does generative AI dependence foster creativity? Evidence from sports undergraduates

**DOI:** 10.1371/journal.pone.0341277

**Published:** 2026-02-09

**Authors:** Weicheng Gu

**Affiliations:** School of Education, Nanchang Vocational University, Nanchang, Jiangxi, China; Gannon University, UNITED STATES OF AMERICA

## Abstract

As an emerging technological tool, generative AI profoundly reshapes higher education teaching and learning processes. Creativity, as a key component of university students’ core competencies, is significantly influenced by technological transformations. However, the mechanisms through which generative AI dependence affects creativity remain underexplored, especially among students majoring in sports. Grounded in Flow Theory, this study investigates the impact of generative AI dependence on the creativity of university students in sports-related disciplines. Using a snowball sampling strategy, the researchers surveyed 453 undergraduates in sports, assessing their self-reported responses across four constructs: generative AI dependence, self-efficacy, flow, and creativity. Partial Least Squares – Structural Equation Modeling was employed to uncover the complex relationships between generative AI dependence and creativity. The results reveal that generative AI dependence has a significant positive direct effect on creativity and an indirect effect mediated by self-efficacy. However, the mediating role of flow was not statistically significant. Furthermore, the multi-group analysis indicates that the positive effects of generative AI dependence on flow and creativity are more pronounced among male students. These findings offer theoretical insights and practical guidance for using generative AI to enhance creativity among students in sports programs.

## Introduction

The rapid advancement of generative AI has profoundly reshaped various aspects of higher education [1]. According to a recent survey, since the release of ChatGPT, 63% of 116 research universities in the United States have encouraged the integration of generative AI into teaching, 41% have provided implementation guidelines, 56% have offered syllabus templates, and 50% have shared relevant course case studies [2]. While this emerging technology has enhanced learning efficiency [3], its extensive application has also intensified sports students’ dependence on AI [4]. Such overreliance may adversely affect students majoring in sports in several critical dimensions, including their capacity for critical thinking in athletic contexts [5], problem-solving abilities in sports instruction and training [6], and creativity in sports innovation and tactical design [7,8]. However, existing research offers limited insight into the underlying mechanisms linking generative AI dependence to creativity among sports students. There is a lack of systematic theoretical explanation and empirical data specifically targeting this unique population. Furthermore, the concept of “Zero Gravity” proposed by Glebova and López-Carril [9] vividly depicts the sense of weightlessness and ambiguity students experience in uncertain and rapidly changing environments, highlighting sports undergraduates as a domain-specific population whose learning processes are particularly sensitive to sector-level disruptions and digital transformation. Additionally, creativity is fundamental in sports training and performance, as students must constantly generate novel tactical ideas and movement adaptations in dynamic sporting environments [10]. Therefore, it is imperative to explore how generative AI dependence influences students’ creativity in sports-related majors and to identify the internal psychological mechanisms driving this relationship.

In the fields of art [11,12], education [13,14], and business [15,16], a growing body of literature has explored how generative AI supports, enhances, or transforms the manifestation and cultivation of human creativity. These discussions have also raised concerns regarding overreliance on generative AI and associated ethical considerations. However, within the domain of physical education, existing research has primarily focused on the technical applications of generative AI, such as customized instructional design [17] and personalized learning experiences [18]. Most studies have adopted frameworks such as the Technology Acceptance Model [19] and Theory of Creativity [20], emphasizing the direct effects of perceived usefulness, ease of use, and frequency of use on creativity. Nevertheless, these studies largely overlook the deeper psychological mechanisms, such as the role of generative AI dependence in shaping self-efficacy and creativity among sports students, which this study aims to investigate.

Although prior research has made progress in exploring the relationship between generative AI and creativity, the generative AI dependence—as a more profound manifestation of technology usage—has not received adequate scholarly attention, and its specific impact on creativity (whether facilitative or inhibitory) remains unclear [21]. To provide a clearer conceptual basis, the present study conceptualizes generative AI dependence as a higher-order construct composed of four distinct and non-interchangeable usage patterns—reflective, cautious, thoughtless, and collaborative—which jointly form students’ overall level of reliance on generative AI. Moreover, existing studies have paid little attention to gender differences, often relying on single-group linear analyses that limit their ability to uncover moderating mechanisms. In particular, the distinctive characteristics of students majoring in sports—such as their learning patterns and technology use—have been largely overlooked, which may obscure how gender moderates the relationship between technology dependence and creativity. Given these theoretical and methodological gaps, a systematic investigation of the relationship between generative AI dependence and creativity among university students in sports programs holds both significant theoretical relevance and practical implications.

Accordingly, this study aims to comprehensively investigate how generative AI dependence influences the creativity of university students majoring in sports, focusing on the mediating roles of self-efficacy and flow, as well as examining potential gender differences in these relationships. The findings are expected to optimize the pedagogical integration of generative AI in sports-related curricula and offer theoretical insights and practical strategies for enhancing creativity among this unique student population. Based on the identified gaps and research needs, the study addresses the following research questions:

1. Does generative AI dependence significantly influence the creativity of sports majors?
2. Does generative AI dependence affect creativity indirectly through psychological constructs such as self-efficacy and flow?
3. Are there significant gender differences in the impact of generative AI dependence on creativity among sports majors?

This study makes three potential contributions to the existing body of knowledge. First, it is the first to introduce the construct of generative AI dependence into creativity research among university students majoring in sports-related disciplines. By doing so, it fills an important gap in the current literature. Second, the study uncovers the mediating role of self-efficacy in the relationship between generative AI dependence and creativity, thereby extending the understanding of how technological engagement shapes creative outcomes within sports education. Third, it identifies gender as a critical moderating factor in the relationship between generative AI dependence and creativity, thereby enriching the understanding of boundary conditions and group-specific differences in creativity research.

## Literature review

### Flow theory

Flow theory was first proposed by Mihaly [22], who posited that individuals enter a state of “flow” when fully immersed and intensely focused in an activity where challenges and personal skills are in balance. This state is characterized by deep absorption, a sense of selflessness, and intense enjoyment, all of which contribute to enhanced creativity and improved task performance. The theory comprises nine dimensions, among which the balance between challenge and skill and focused attention are considered core components [22]. Specifically, the challenge-skill balance refers to a dynamic equilibrium in which the perceived difficulty of a task closely matches the individual’s capabilities; focused attention denotes the complete concentration on the task at hand, effectively filtering out irrelevant distractions [22]. These two dimensions are closely interrelated: when the challenge level of an activity aligns with an individual’s skill set, it facilitates sustained attention; conversely, heightened concentration enables individuals to coordinate the relationship between challenge and skill better, making it easier to achieve a state of flow. This study investigates the influence of generative AI dependence on creativity among university students majoring in sports. Given that flow theory emphasizes the role of focused attention and the balance between skills and challenges in fostering creativity, and that dependence on generative AI may disrupt this balance, the theory offers a valuable framework for understanding how generative AI dependence may impact the creative processes of sports students. Therefore, flow theory is considered highly applicable in the context of this research.

Flow theory has recently been widely applied across various research domains, particularly in studying user behavior within digital technology. In augmented reality, Brannon Barhorst, McLean, Shah and Mack [23] found that augmented reality experiences elicit stronger flow states than traditional shopping experiences. In AI contexts, Poushneh [24] demonstrated that the personality traits of voice assistants can drive users’ flow experiences during voice interactions, influencing their attitudes and behavioral intentions. In streaming media, Tian and Frank [25] revealed that interactivity, usefulness, and entertainment value stimulate viewers’ flow experiences, shaping user engagement behaviors. These studies collectively indicate that flow theory possesses strong explanatory power across diverse contexts; however, the intensity and formation mechanisms of flow experiences vary across different user groups and situational settings.

Moreover, as academic research has advanced, flow theory has increasingly been integrated with other theoretical frameworks to enhance its explanatory and predictive power. For instance, Kumari, Bala and Chakraborty [26] combined the Uses and Gratifications Theory with Flow Theory to identify ten factors influencing consumers’ intentions to use metaverse platform services. Their study also identified three primary types of gratification and flow experiences associated with using these platforms, confirming that the integrated theoretical model offered greater explanatory power than a single-theory approach. Similarly, Wang, Sun, Zhang, Zhang, Feng and Morrison [27] integrated Flow Theory with the Stimulus–Organism–Response framework to examine the impact of virtual reality applications on museum tourism. Their findings revealed the intricate relationships among exhibition formats, flow experiences, and virtual reality technologies, thereby improving the accuracy of behavioral predictions in user engagement. These integrated theoretical approaches underscore the high compatibility and flexibility of Flow Theory in adapting to and explaining increasingly complex user behavior phenomena. However, such integrations can also introduce challenges, including conceptual ambiguity or overlap, which complicates empirical validation. Additionally, integrated models’ explanatory power and generalizability may be highly context-dependent, limiting their transferability to other research settings.

Despite the expanding application of flow theory and its significant research contributions, several limitations remain in the existing literature. First, most studies have focused on general user populations. At the same time, insufficient theoretical attention has been paid to the behavior of technology usage, flow experiences, and creativity mechanisms of highly specialized groups such as university students majoring in sports. Second, the complex mechanisms underlying the relationship between flow and creativity have not been clearly articulated, and a unified theoretical framework to explain their dynamic causal links is still lacking. Given the unique characteristics of sports students in terms of flow-inducing conditions, patterns of technological dependence, and creative performance, exploring the relationship between their dependence on generative AI and creativity can offer a valuable extension to current flow theory perspectives. This study seeks to address this theoretical gap by systematically analyzing the types of generative AI dependence among sports students and examining their specific impact on creativity. It aims to provide new theoretical insights and empirical evidence contributing to flow theory’s conceptual refinement and practical application.

### Creativity

Creativity, a complex psychological and social phenomenon, has long been recognized as a key capability driving educational reform, technological advancement, and individual development [28]. In recent years, with the widespread adoption of digital education and AI technologies, the study of creativity has expanded beyond controlled laboratory settings to encompass more complex real-world contexts such as higher education [29], AI usage [30], organizational management [31], and physical education [32]. Increasingly, research has focused on the dynamic mechanisms through which creativity emerges at the intersection of technology, context, and individual characteristics.

Specifically, research on the creativity of students majoring in sports is still in its early stages. Sports undergraduates constitute a domain-specific population whose learning behaviors, skill development, and career trajectories are shaped by the evolving expectations of sport education and the dynamic nature of the sport industry [9]. These domain-specific characteristics may contribute to unique patterns in the relationship between generative AI dependence, self-efficacy, and creativity, distinguishing sports students from those in other academic disciplines. As a highly practice-oriented academic group, sports students often develop creative expression through skill training and physical performance [33]. However, in the growing prevalence of generative AI, this group has shown an increasing tendency to rely on generative AI for design assistance, feedback suggestions, and content generation. Such dependence may alter their original cognitive processing and creative development processes. The potential pathway linking “technological dependence – psychological motivation – creative output” remains theoretically underexplored and lacks systematic empirical validation.

Existing studies have explored creativity through various theoretical frameworks, including the Meta-Creativity Model [29], Socio-Technical Systems Theory [34], Self-Determination Theory [35], and Flow Theory [31]. Among these, flow theory is one of the most commonly employed. It is particularly valued for its focus on immersive experiences, which is especially relevant for understanding collaborative creativity supported by generative AI. For example, Kulichyova, Ali, McCracken, Woods and Moffett [31] found that the flow state can enhance individuals’ self-awareness, allowing them to recognize their creative potential and thereby stimulate creativity. While Flow Theory highlights the positive role of immersive experiences in activating creative potential, it tends to overlook the nonlinear nature of creative processes, the complexity of socio-technical contexts, and the inherent tensions within cognitive mechanisms.

Finally, from the perspective of underlying mechanisms, the development of creativity is influenced by a combination of technological, psychological, and social factors. On the technological level, features of generative AI—such as information diversity [36], prompt feedback, and interactivity [37,38]—are considered conducive to stimulating idea generation. For instance, Li, Kim and Palkar [37] demonstrated that virtual reality, by providing a highly interactive and dynamic learning environment, enables students to freely explore various solutions and solve challenging tasks, thereby enhancing their creativity. However, most studies on technology emphasize the general functionality of tools and overlook the unique attributes of intelligent technologies like generative AI and the deep interaction mechanisms between such technologies and psychosocial factors. This limits the ability to explain the complex generative process of creativity in contexts where technology is deeply embedded. On the psychological level, variables such as self-efficacy [39], psychological capital [40], immersive experience [31], and psychological safety [41] are significantly and positively associated with creativity. For example, Manik, Sidharta, Zulfikar, Rahman, Fitria, Resawati and Nurdiansyah [40] found that psychological capital enhances individual curiosity, indirectly increasing creativity. However, psychological research often centers on internal individual mechanisms while neglecting the synergistic influence of technological and social contexts, limiting the theoretical explanatory power and practical applicability of such findings. On the social level, factors such as social norms [42], organizational culture [43], social networks [44], and educational support systems [45] are regarded as part of the external support system that shapes individual creative tendencies. For example, Du, Zhang, Zhang, Zhang and Chen [42] found that descriptive and injunctive norms enhance creativity at both individual and societal levels through behavioral guidance, motivational pressure, and social identification. Nevertheless, while social factors emphasize external environments and interpersonal interactions, they often neglect the foundational roles of individual psychological mechanisms and technological conditions. This leads to insufficient explanations of how creativity originates at the individual level and is catalyzed by technology. In summary, existing research lacks a comprehensive understanding of the mechanisms underlying the creativity of sports students, particularly in contexts characterized by deep technological integration and the interaction of psychological and social factors. This study is the first to systematically introduce “generative AI dependence” as a key variable in this field, aiming to uncover the complex psychological mechanisms that shape creativity in generative AI dependence.

## Hypotheses

### Generative AI dependence and creativity

Generative AI dependence refers to users’ excessive reliance on AI-generated content, suggestions, or solutions to complete tasks related to learning, work, or decision-making [46]. Accumulating empirical evidence suggests that generative AI dependence plays a critical role in shaping creative outcomes, although its effects are not uniform. [4,47]. For example, drawing on Creativity Theory, Alzubi, Nazim and Alyami [47] found that ChatGPT enhances students’ creativity by providing writing inspiration, grammar and vocabulary feedback, and simulated dialogue practice opportunities. Zhang and Xu [4] further demonstrated that the frequency of generative AI usage directly affects technological dependence and indirectly shapes it through perceived efficiency and confidence. However, recent empirical studies have cautioned that excessive or automation-oriented dependence on generative AI may undermine creativity by inducing cognitive passivity, information overload, or long-term creative erosion [48–50]. At the same time, emerging evidence indicates that generative AI dependence can foster creative performance when it facilitates effective cognitive resource allocation and engagement in creative processes under appropriate conditions [51]. According to Cognitive Load Theory, moderate dependence on generative AI can effectively reduce cognitive load, freeing up mental resources for higher-order thinking and creative processes [52]. As creativity is widely recognized as a key learning outcome in sports education [53], and can be influenced by technological facilitation through internal cognitive and motivational pathways, it is conceptualized as the outcome variable in the proposed model [54]. Cognitive Load Theory suggests that when generative AI supports lower-level analytical processing, students can focus more cognitive resources on higher-order tactical thinking [55]. In dynamic sports learning environments where creativity depends on rapid strategic adaptation, such resource reallocation may enhance sports-major undergraduates’ creative performance. Based on these theoretical foundations and prior empirical findings, this study proposes the following hypothesis:

H1: Generative AI dependence significantly and positively predicts creativity among university students majoring in sports.

### Generative AI dependence, self-efficacy, and creativity

Self-efficacy refers to an individual’s belief in their ability to successfully perform specific tasks or cope with particular situations [56]. Prior research has shown that dependence on generative AI significantly predicts self-efficacy [4,57]. For instance, drawing on Self-efficacy Theory and Social Cognitive Theory, Buniel, Intano, Cuartero, Grustan, Sumaoy, Reyes, Calipayan, Arreo, Duero, Rosil, Agustin, Diron, Pingol, Sapuras, Miranda, Julve, Josol, Mercado, Latoja, Cubillan, Fallado, Duran, Ambray, Miranda, Etchon, Ramoso, Rubenial, Ganancias, Orozco, Gracia, Notado, Darao and Cortes [57] argued that generative AI dependence enhances students’ self-efficacy by simplifying tasks and enriching the learning experience. Furthermore, based on Technology Dependence Theory, Zhang and Xu [4] found that more frequent use of generative AI significantly increases students’ self-efficacy, including their perceived problem-solving efficiency and confidence. And social cognitive theory proposes that performance assistance strengthens self-efficacy by reinforcing mastery experiences [58]. Therefore, AI-enabled guidance in sport-specific tasks may particularly heighten students’ confidence in their athletic and tactical abilities. Building on these theoretical foundations and empirical findings, the present study proposes the following hypothesis:

H2: Generative AI dependence significantly and positively predicts self-efficacy among university students majoring in sports.

Previous studies have consistently demonstrated that self-efficacy is a key predictor of creativity [59–61]. For example, based on Self-efficacy Theory, Haase, Hoff, Hanel and Innes-Ker [59] found that self-efficacy fosters creativity by enhancing individuals’ motivation. Similarly, Tierney and Farmer [61] proposed that self-efficacy indirectly influences creativity by shaping employees’ motivation, emotions, and behaviors. Building on these findings, the present study proposes the following hypotheses:

H3: Self-efficacy significantly and positively predicts creativity among university students majoring in sports.
H4: Self-efficacy mediates the relationship between generative AI dependence and creativity among university students majoring in sports.

### Generative AI dependence, flow, and creativity

Flow refers to a highly immersive and enjoyable psychological state experienced when individuals fully engage in an activity [62]. Prior research has identified dependence on generative AI as a significant predictor of flow [63–65]. For instance, based on Flow Theory, Shi, Li and Zhang [64] found that generative AI dependence enhances learners’ flow experiences by providing highly personalized learning environments that facilitate a balance between skill and challenge. Similarly, Juvina, O’Neill, Carson, Menke, Wong, McNett and Holsinger [65] demonstrated that generative AI supports real-time task difficulty adjustments, helping users maintain this balance and promoting flow states. Flow Theory emphasizes the balance between challenge and skill as a prerequisite for immersive engagement; AI-supported skill adjustment may facilitate such balance in physical-practice-oriented learning, promoting flow experiences relevant to creative sport performance. Drawing on the above literature, this study proposes the following hypothesis:

H5: Generative AI dependence significantly and positively predicts flow among university students majoring in sports.

Previous studies have shown that flow is a significant determinant of creativity [66–68]. For example, drawing on Interest and Flow Theories, Dan [69] investigated the relationships among high school students’ learning interest, flow, and creativity. The results revealed that flow indirectly promotes creativity by enhancing students’ interest in learning and fostering positive emotional experiences. Additionally, based on Flow Theory and Creativity Theory, Zaman, Anandarajan and Dai [70] found that flow does not directly influence creativity but enhances it indirectly through exploratory behaviors and positive emotions. Building on these findings, this study proposes the following hypotheses:

H6: Flow significantly and positively predicts creativity among university students majoring in sports.
H7: Flow mediates the relationship between generative AI dependence and creativity among university students majoring in sports.

### Self-efficacy and flow

Previous research has established that self-efficacy is an important antecedent of flow [71–73]. For example, Jia, Meng, Ma and Mao [71] suggested that self-efficacy influences learners’ cognitive assessment of the match between their abilities and task challenges, enhancing their sense of control over the learning process and ultimately facilitating the flow experience. Additionally, drawing on Social Cognitive Theory and Flow Theory, Peifer, Schönfeld, Wolters, Aust and Margraf [73] found that self-efficacy promotes the emergence of flow by strengthening students’ confidence in their own abilities. Based on these findings, this study proposes the following hypotheses:

H8: Self-efficacy significantly and positively predicts flow among university students majoring in sports.
H9: Self-efficacy and flow sequentially mediate the relationship between generative AI dependence and creativity among university students majoring in sports.

### The moderating role of gender

According to Social Role Theory, societal expectations and behavioral norms related to gender roles influence individuals’ use of technology and how their creativity is expressed [74,75], especially in sports contexts where achievement expression and risk-taking tendencies differ between males and females [76]. Although current research has not explicitly addressed gender differences in the relationship between dependence on generative AI and creativity, studies have shown that males use generative AI at significantly higher rates than females [77], which may impact their creative performance [78]. In addition, prior research has found significant gender differences in creative self-efficacy, with male students reporting higher levels than their female counterparts [79]. Gender differences have also been observed in flow experiences; for example, Sigmundsson and Leversen [80] found that male students scored higher in flow than female students. Based on these theoretical insights and empirical findings, this study proposes the following hypotheses:

H10: The effect of generative AI dependence on creativity differs significantly by gender among university students majoring in sports.
H11: The effect of generative AI dependence on self-efficacy differs significantly by gender among university students majoring in sports.
H12: The effect of generative AI dependence on flow differs significantly by gender among university students majoring in sports.

**Fig 1:**
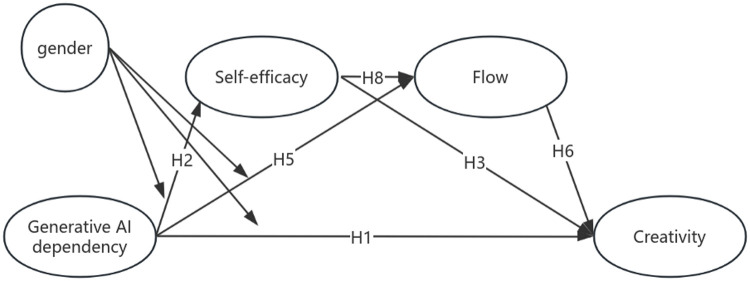
Hypothesized Model.

Based on the above hypotheses, the conceptual model of this study is illustrated in Fig 1.

## Method

### Sample and data

Before completing the questionnaire, participants were provided with detailed information about the study, including its purpose and measures for protecting participant confidentiality. They were allowed to proceed with the survey only after providing written informed consent and were informed of their right to withdraw without penalty. Data was collected from March 12 to April 18, 2025, using the Chinese online survey platform Wenjuanxing (https://www.wjx.cn/). This study employed a snowball sampling method to recruit participants. This approach is particularly suitable for specific populations, such as sports majors, who exhibit a degree of internal homogeneity and possess established social networks [81]. Its strength lies in the ability to efficiently and cost-effectively reach various sub-groups (e.g., different sport specialties) through initial “seed” participants. Furthermore, peer referrals help establish initial trust, facilitating the collection of more authentic and in-depth data. We acknowledge that this method may affect the generalizability of the findings. To enhance the reliability of the study, data collection proceeded until the pre-determined sample size target was reached. This target was set to ensure adequate statistical power for model testing and to capture the diversity within the population of sports majors across different specialties. Additionally, Wenjuanxing’s built-in response time tracking feature was used to monitor and assess data quality. Respondents who completed the survey with valid answers were offered a random cash incentive as compensation for their participation.

After completing all necessary preparations, data were collected from 518 undergraduate students at three universities of Jiangxi Province, China. To ensure data quality, the study adopted a three-step screening process based on the approach proposed by Wu, Li, Zheng and Guo [82]. First, a pilot test indicated that participants took between 2 and 5 minutes to complete the survey under normal conditions. Respondents who completed the questionnaire in less than 90 seconds were considered to have rushed through irresponsibly, and their responses were deemed invalid. Second, the questionnaire included a reverse-coded item; participants who failed to respond appropriately to this item were assumed to be inattentive and were excluded. Third, any responses with identical answers were also removed from the dataset. Following this rigorous screening process, 65 invalid responses were excluded, resulting in a final sample of 453 valid responses for further analysis. The maximum number of arrows pointing to any endogenous latent construct in the proposed research model is three. According to Hair [83], to achieve a minimum R^2^ of 0.10 with 1% significance level, the required minimum sample size is 124. Therefore, the final sample of 453 participants far exceeds this threshold, ensuring the robustness and reliability of the study’s findings.

Among the 453 valid responses, 295 participants were male (65.1%) and 158 were female (34.9%). In terms of academic year, 136 were freshman (30.0%), 158 were sophomore (34.9%), and 152 were junior (33.6%). Detailed demographic information is presented in Table 1. This representative sample provides a solid foundation for analyzing the impact of generative AI dependence on the creativity of university students majoring in sports. The diversity and size of the sample enhance the generalizability of the findings within this research.

**Table 1:** Demographic Characteristics (N = 453).

Demographics	Category	Frequency	Percentage
Gender	Male	295	65.1%
	Female	158	34.9%
Grade	Freshman	136	30.0%
	Sophomore	158	34.9%
	Junior	152	33.6%
	Senior	7	1.5%
Age	18-21	357	78.8%
	22-25	93	20.5%
	25 above	3	0.7%
Usage frequency	1-2 times/day	226	49.9%
	3-5 times/day	147	32.5%
	Over 5 times/day	80	17.6%

### Measurements

The measurements was divided into two main sections. The first section collected participants’ demographic information, while the second section gathered self-reported data on each construct examined in the study. Validated scales were employed to assess these constructs, and the items were appropriately adapted to align with this research’s specific context and objectives. This approach ensured that the responses accurately reflected participants’ perceptions and experiences within the study.

In addition to collecting basic demographic information, the measurements included four key constructs: generative AI dependence, self-efficacy, flow, and creativity. Compared to the 7-point Likert scale, the 5-point Likert scale has been shown to offer significant advantages in improving reliability and validity [84], minimizing response bias, and enhancing the sensitivity of statistical analysis [85]. Therefore, all constructs in this study were measured using 5-point Likert scales, ranging from (1) “Strongly Disagree” to (5) “Strongly Agree.”

To ensure conceptual and semantic equivalence between the English original scales and their Chinese versions used in this study, a rigorous translation and back-translation procedure was implemented. The process involved the following steps: (1) Forward translation by a bilingual researcher; (2) Synthesis and review by the research team; (3) Back-translation by an independent bilingual expert unfamiliar with the original scales; and (4) Iterative comparison and refinement until consensus was achieved. The final Chinese items are provided in the supplementary materials.

The Generative AI dependence Scale, adapted from Hou, Zhu, Sudarshan, Lim and Ong [86], assesses the degree to which university students majoring in sports-related disciplines rely on generative AI tools during daily learning activities and task completion. In the original scale development study, Hou, Zhu, Sudarshan, Lim and Ong [86] established the underlying factor structure through exploratory procedures and reported satisfactory psychometric properties for this scale. The scale comprises four dimensions: reflective use (e.g., “I critically evaluate the output generated by AI tools”, 4 items), cautious use (e.g., “I recognize that the outputs generated by AI tools are not perfect”, 4 items), thoughtless use (e.g., “I directly copy AI-generated content into my solutions without modification”, 4 items), and collaborative use (e.g., “My peers help me revise the output produced by generative AI tools”, 4 items). The Cronbach’s alpha for the four dimensions are 0.774, 0.833, 0.857, and 0.878, respectively. The overall Cronbach’s alpha for the scale is 0.736, indicating satisfactory internal consistency.

The Creativity Scale, adapted from Tan and Ong [87], is intended to assess the ability of university students majoring in sports-related disciplines to demonstrate originality and uniqueness in their thinking during learning and practical activities. The scale consists of five items (e.g., “With the help of generative AI, I can propose new ways to achieve my goals”). It has a Cronbach’s alpha of 0.908, indicating excellent internal consistency reliability.

The Flow Scale, adapted from Bakker and van Woerkom [88], measures whether university students majoring in sports-related disciplines experience a highly focused and enjoyable “flow” state while using generative AI to support their learning or training. The scale comprises four items (e.g., “When interacting with generative AI, I enter a state of complete absorption”) and demonstrates excellent internal consistency, with a Cronbach’s alpha of 0.919.

The Self-Efficacy Scale, adapted from Fangzhou, Lin and Lai [89], is designed to assess the perceived ability of university students majoring in sports-related disciplines to complete tasks and solve problems effectively. The scale includes three items (e.g., “I believe I can complete required tasks more effectively with the help of generative AI”). It demonstrates good internal consistency, with a Cronbach’s alpha of 0.803. See Table S1 in S1 Appendix for the detailed questionnaire items.

### Data analysis

This study employed Partial Least Squares- Structural Equation Modeling (PLS-SEM) to examine the linear relationships between exogenous and endogenous variables. Following established guidelines for variance-based structural equation modeling, measurement validity in this study was assessed through indicator reliability, internal consistency reliability, convergent validity, and discriminant validity within the measurement model. As this research adopted a theory-driven, confirmatory PLS-SEM approach, exploratory factor analysis (EFA) was not conducted, in line with established PLS-SEM guidelines [90]. PLS-SEM offers significant advantages, particularly in small sample sizes, non-normal data distributions, exploratory research, and complex model structures [83]. With a sample size of only 453 and a model comprising four constructs and 28 items, this study examines a relatively complex theoretical model using a variance-based PLS-SEM approach [91]. Therefore, PLS-SEM is deemed appropriate for the data analysis in this research. According to Hair [83], PLS-SEM analysis consists of two key components: the measurement and structural models. The measurement model assessment includes scale reliability, convergent validity, and discriminant validity tests. The structural model assessment involves evaluating multicollinearity, path significance, explanatory power, and conducting multi-group analyses.

### Ethics statement

This study was reviewed and approved by the Human Research Ethics Committee of Nanchang Vocational University (Approval No. NVU-2025-01-0001). The study complies with the National Statement on Ethical Conduct in Human Research (2007). Written informed consent was obtained from all participants prior to their involvement in the research.

## Results

The researchers employed various statistical techniques to develop and validate the research findings. Hair [92] distinguished between the first and second generations of statistical methods. First-generation techniques, such as factor and regression analyses, were widely used and dominated earlier research. Since the 1990s, more sophisticated multivariate methods—such as Structural Equation Modeling—have emerged, becoming the cornerstone of second-generation statistical approaches [93]. Structural Equation Modeling is typically categorized into two types: covariance-based and variance-based. Given the complexity of the model in this study—comprising four constructs and 28 items—variance-based Structural Equation Modeling, specifically PLS-SEM, was deemed appropriate [92]. The analysis used Smart Partial Least Squares 4.0 to evaluate the measurement and structural models.

### Measurement model

The evaluation of the measurement model followed established criteria:

Step 1: Indicator Reliability: The outer loadings of each indicator should be equal to or greater than 0.707, indicating acceptable reliability [92].
Step 2: Internal Consistency Reliability: Two metrics assessed internal consistency: Cronbach’s alpha (α) and Composite Reliability (CR). For both indicators, a threshold value of ≥ 0.70 is recommended [94].
Step 3: Validity

(1) Convergent Validity: Convergent validity is assessed using the Average Variance Extracted (AVE), which should be equal to or greater than 0.50 to indicate an adequate level of shared variance among items measuring the same construct [95].
(2) Discriminant Validity: Discriminant validity was evaluated using two established methods:

First, the Fornell–Larcker criterion (Fornell & Larcker, 1981), which compares the AVE square root for each construct with its correlations with other constructs.

Second, the Heterotrait–Monotrait Ratio of Correlations (HTMT) [96] is considered a more robust technique for assessing discriminant validity in variance-based Structural Equation Modeling.

Following Hair [92] recommendation, indicator outer loadings should exceed 0.707. In this study, the outer loadings of 16 items met this criterion. However, one indicator under the Thoughtless Use dimension of the generative AI dependence exhibited an outer loading slightly below 0.70. According to Hair [83], indicators with loadings between 0.40 and 0.70 may be retained if both the AVE and CR for the construct meet the required thresholds. Therefore, the item was retained in the model.

Next, internal consistency reliability and convergent validity were assessed using Cronbach’s alpha, CR, and AVE. As shown in Table 2, all values met the minimum thresholds for indicator reliability and internal consistency. In addition, the AVE values for all constructs were equal to or greater than 0.50, indicating satisfactory convergent validity.

**Table 2:** Reliability and Average Variance Extracted.

Constructs	Items	Outer loadings	Cronbach’ α	CR	AVE
CRE	CRE1	0.825	0.908	0.909	0.731
	CRE2	0.834			
	CRE3	0.877			
	CRE4	0.872			
	CRE5	0.864			
FL	FL1	0.840	0.919	0.923	0.805
	FL2	0.926			
	FL3	0.922			
	FL4	0.897			
SE	SE1	0.880	0.803	0.807	0.72
	SE2	0.883			
	SE3	0.778			
GAI DEP	Thoughtless use	0.622	0.736	0.743	0.562
	Cautious use	0.784			
	Collaborative use	0.806			
	Reflective use	0.772			

*Note: GAI DEP-Generative AI dependency; CRE-Creativity; FL-Flow; SE-Self-efficacy;*

Third, Table 3 presents the correlation matrix for the Fornell–Larcker discriminant validity test. According to Hair [83], the square root of the AVE for each construct should be greater than its highest correlation with any other construct in the model. The results satisfy this criterion, indicating acceptable discriminant validity based on the Fornell–Larcker criterion.

**Table 3:** Discriminant Validity (Fornell-Larcker Criteria).

	Generative AI dependency	Creativity	Flow	Self-efficacy
GAI DEP	**0.750**			
CRE	0.683	**0.855**		
FL	0.375	0.372	**0.897**	
SE	0.693	0.687	0.372	**0.848**

Note: The bolded values on the diagonal represent each construct’s square roots of the Average Variance Extracted.

The Heterotrait–Monotrait Ratio of Correlations, proposed by Henseler, Ringle and Sarstedt [96], is a robust criterion for assessing discriminant validity. The Heterotrait–Monotrait is calculated as the mean of the heterotrait–heteromethod correlations relative to the mean of the monotrait–heteromethod correlations. Heterotrait–heteromethod correlations refer to correlations between indicators across different constructs, while monotrait–heteromethod correlations refer to correlations among indicators within the same construct. The Heterotrait–Monotrait values were computed using Smart Partial Least Squares software and are presented in Table 4. All values fall within the acceptable threshold of ≤ 0.90, as recommended by [96], thereby confirming discriminant validity.

**Table 4:** Discriminant Validity (The Heterotrait–Monotrait Criteria).

	Generative AI dependency	Creativity	Flow	Self-efficacy
GAI DEP				
CRE	0.827			
FL	0.442	0.407		
SE	0.890	0.801	0.436	

### Structural model

The evaluation of the structural model followed the guidelines proposed by Hair [83] and involved the following steps:

(1) Assessment of Collinearity. Variance Inflation Factor (VIF) values were examined to detect potential multicollinearity issues. First, the inner VIF values were assessed for the reflective measurement models. Subsequently, to provide a comprehensive evaluation of the structural model, the Full Collinearity VIF (FVIF) was employed [97]. VIF and FVIF values below 3.3 indicate no critical collinearity concerns [98].
(2) Assessment of Path Coefficient Significance. The statistical significance of the relationships between constructs was evaluated using *p*-values. A threshold of *p* < 0.05 was used to determine significance.
(3) Assessment of the Coefficient of Determination (R^2^). The R^2^ values were used to evaluate the model’s explanatory power. As a rule of thumb, R^2^ values of 0.190, 0.333, and 0.670 are considered weak, moderate, and substantial, respectively.

First, collinearity was assessed using a two-pronged approach. The inner VIF values for the reflective measurement models were examined, as summarized in Table 5, and the Full Collinearity VIF (FVIF) values for the structural model were evaluated, as presented in Table 6. All inner VIF and FVIF values were below the conservative threshold of 3.3, indicating that multicollinearity is not a concern in this study.

**Table 5:** Variance Inflation Factor.

	Generative AI dependency	Creativity	Flow	Self-efficacy
GAI DEP		1.983	1.923	1.000
CRE				
FL		1.197		
SE		1.978	1.923	

**Table 6:** Full Collinearity VIF.

	Generative AI dependency	Creativity	Flow	Self-efficacy
GAI DEP		1.983	2.280	1.936
CRE	1.950		2.243	1.932
FL	1.196	1.197		1.199
SE	1.949	1.978	2.300	

**Fig 2:**
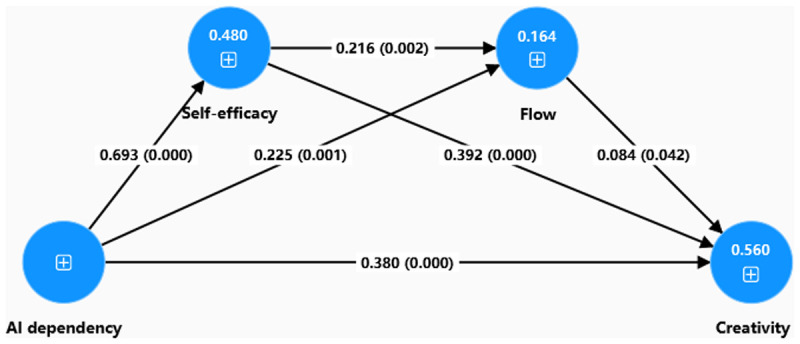
Path coefficients of Structural model.

Second, the path coefficients (β values) representing the relationships between constructs in the model are presented in Table 7. The significance of the path coefficients was assessed using the bootstrapping procedure in Partial Least Squares. This analysis was conducted with 5,000 bootstrap subsamples to generate stable estimates, and 95% confidence intervals were examined to robustly test the significance of each path. Fig 2 presents the analysis results of this structural model. Both *t*-values and *p*-values were used to determine whether the path coefficients were statistically significant at the 5% level. Specifically, statistical significance at the 5% level requires *p* < 0.05 and *t* > 1.96. The results of the bootstrapping analysis are summarized in Table 7. Among the direct predictors of creativity, self-efficacy had the strongest effect (β = 0.392, *t* = 7.008, *p* = 0.000), followed by generative AI dependence (β = 0.380, *t* = 6.726, *p* = 0.000), and flow (β = 0.084, *t* = 2.034, *p* = 0.042). Additionally, generative AI dependence significantly predicted flow (β = 0.225, *t* = 3.239, *p* = 0.001) and self-efficacy (β = 0.693, *t* = 19.114, *p* = 0.000). Finally, self-efficacy was also found to influence flow significantly (β = 0.216, *t* = 3.143, *p* = 0.002).

**Table 7:** Results of Hypothesis Testing.

Hypothesis	β	2.50%	97.50%	T statistics	P values	Results
GAI DEP → CRE	0.380	0.269	0.490	6.726	0.000	Supported
GAI DEP → FL	0.225	0.091	0.361	3.239	0.001	Supported
GAI DEP → SE	0.693	0.617	0.759	19.114	0.000	Supported
FL → CRE	0.084	0.003	0.166	2.034	0.042	Supported
SE → CRE	0.392	0.282	0.498	7.008	0.000	Supported
SE → FL	0.216	0.079	0.346	3.143	0.002	Supported

Third, the coefficient of determination (R^2^) represents the proportion of variance in an endogenous construct explained by all associated exogenous constructs [83]. Values around 0.67 are considered substantial, around 0.33 moderate, and around 0.19 weak. As shown in Table 8, generative AI dependence, flow, and self-efficacy collectively explained 56.0% of the variance in creativity, indicating a moderate to substantial explanatory power. To evaluate the predictive relevance of the model, we calculated the Stone-Geisser’s Q^2^ value through a blindfolding procedure. Q^2^ > 0 suggests that the model has predictive power [83]. As presented in Table 8, the Q^2^ value for all constructs were greater than 0, confirming the model’s substantial predictive relevance. Furthermore, we examined the effect size (f^2^) to determine the substantive impact of each exogenous construct on the endogenous constructs. Following Cohen’s guidelines [99], f^2^ values of 0.02, 0.15, and 0.35 represent small, medium, and large effects, respectively. The f^2^ values in Table 8 indicate that the construct of Self-efficacy (f^2^ = 0.923) had a large effect on generative AI dependency.

**Table 8:** The predictive power.

Constructs	R^2^	f ^2^	Q^2^
CRE	0.560	0.166	0.404
FL	0.164	0.032	0.129
SE	0.480	0.923	0.342

### Mediation analysis

Mediation analysis assessed the mediating roles of self-efficacy and flow in the relationship between generative AI dependence and creativity. A PLS-SEM mediation analysis was performed using the bootstrapping procedure [100]. The significance of both direct and indirect effects, along with the sign of the product of coefficients, was examined to determine the type and strength of mediation. The results are summarized in Table 9. Findings revealed that self-efficacy significantly mediated the relationship between generative AI dependence and creativity, whereas flow did not mediate. To determine the nature of the mediation, the direct effect between generative AI dependence and creativity was also examined and found to be significant. This indicates that self-efficacy is a partial mediator, while flow does not mediate the relationship between generative AI dependence and creativity. Finally, the serial mediation effect of flow and self-efficacy in the pathway from generative AI dependence on creativity was tested but found to be non-significant, indicating no chain mediation effect exists.

**Table 9:** Mediation Analysis Results.

Relationship	Indirect effect	T values	P values	Direct effect	T values	P values	Intermediary type
Mediation Effect of Self-efficacy							
GAI DEP → SE → CRE	0.271	6.162	0.000	0.380	6.726	0.000	PM
Mediation Effect of Flow							
GAI DEP → FL → CRE	0.019	1,719	0.086	0.380	6.726	0.000	NM
Mediation Effect of Flow and Self-efficacy							
GAI DEP → SE → FL → CRE	0.013	1.621	0.105	0.380	6.726	0.000	NM

Note: NM – No Mediation; PM – Partial Mediation.

### Multi-Group analysis

Multi-group comparison consists of three steps: first, create data groups; then, test for invariance, and finally, assess group difference.

First of all, create a data group, to assess the differences between the different groups. According to the purpose of the study [101], the number of men and women should not differ greatly. However, if the sample size difference between the two groups is more than 50%, the statistical test results may be biased [98]. The number of males and females in this study is 295 and 158, respectively, the difference between the two groups is less than 50%. Therefore, there is no bias in the statistical results.

In the next place, test for invariance. Checking consistency with the MICOM program, Henseler, Ringle and Sarstedt [102]state that the process consists of three steps: configural invariance, compositional invariance, and equality of composite means value and variances. First of all, the establishment of configuration variance needs to follow the following guidelines: (a) identical indicators per measurement model, (b) identical data treatment, and (c) identical algorithm settings or optimization criteriaIn this study, the above criteria are met. Secondly, as suggested by Matthews [103], the compositional invariance test suggests that original correlations should be equal to or greater than the 5.00% quantile correlations. Table 10 shows that the composite variance test of all potential variables meets the criteria. Third, evaluate the equality of composite variables and mean values in each group. For invariance to be established, the first column (mean original difference) must be a number that falls within the 95% confidence interval. Table 11 shows that the first part of the results does not meet the requirements. The results in Table 12 establish that the variance-original difference for all latent variables are consistent with the 95% confidence interval. Therefore, the second part of the result does not meet the requirements. When steps 1 and 2 are satisfied but step 3 is not, partial measurement invariance is provided. In fact, partial measurement invariance is sufficient to perform PLS-MGA to compare the structural paths between groups, allowing for multi-group analysis [102].

**Table 10:** MICOM step 2 result report.

Constructs	Original correlation	5.00%	Permutation p value	Results
GAI DEP	0.994	0.986	0.237	Yes
CRE	1.000	0.999	0.306	Yes
FL	1.000	0.997	0.720	Yes
SE	0.998	0.995	0.230	Yes

**Table 11:** MICOM step 3a result report.

Constructs	Mean-Original difference (male-female)	Mean-Permutationmean difference (male-female)	2.50%	97.50%	Permutation p value	Equal mean values
GAI DEP	0.115	0.000	−0.23	0.232	0.344	Yes
CRE	0.248	0.000	−0.231	0.232	0.037	NO
FL	0.338	−0.001	−0.23	0.228	0.005	NO
SE	0.349	0.001	−0.234	0.232	0.005	NO

**Table 12:** MICOM step 3b result report.

Constructs	Variance-Original difference (male-female)	Variance-Permutation mean difference (male-female)	2.50%	97.50%	Permutation p value	Equal variance values
GAI DEP	0.156	0.032	−0.542	0.64	0.596	Yes
CRE	−0.007	0.024	−0.396	0.456	0.974	Yes
FL	0.233	0.015	−0.265	0.314	0.119	Yes
SE	0.005	0.023	−0.411	0.479	0.985	Yes

Finally, assessment of group differences. This study employed the Partial Least Squares-Multi-Group Analysis method and applied the Welch–Satterthwaite test to examine gender differences in the relationships between sports major undergraduates’ dependence on generative AI and their self-efficacy, flow, and creativity [104]. Table 13 presents the results of the gender-based path difference analysis. The findings indicate that the effects of generative AI dependence on flow and creativity differ significantly between male and female students. However, no significant gender difference was observed in the relationship between generative AI dependence and self-efficacy.

**Table 13:** Multi-Group Analysis.

	Difference (Male-Female)	2.5% (Female)	97.5% (Female)	2.5% (Male)	97.5% (Male)	t value (Male vs Female)	p-value (Male vs Female)
GAI DEP → CRE	0.293	−0.112	0.406	0.322	0.560	2.036	0.044*
GAI DEP → FL	0.441	−0.479	0.248	0.141	0.450	2.184	0.031*
GAI DEP → SE	−0.052	0.568	0.834	0.593	0.758	0.628	0.531

Note: grouped as: male, n = 295; female, n = 158.

## Discussion

This study centers on three core research questions and systematically examines the mechanisms and predictive power of generative AI dependence on the creativity of sports major undergraduates. The discussion proceeds from the main findings and, drawing on Flow theory, provides an in-depth analysis across four dimensions: theoretical mechanisms, variable relationships, gender differences, and theoretical contributions.

The findings reveal that generative AI dependence has a significant positive direct effect on the creativity of sports major undergraduates (p < 0.001). This result is consistent with previous research [47], which emphasizes that generative AI enhances students’ creativity by reducing cognitive load by providing creative inspiration, professional feedback, and interactive support. This effect can be explained by the cognitive resource allocation mechanism in Cognitive Load Theory, which posits that when individuals are freed from basic information-processing tasks, their cognitive resources can be redirected toward higher-order creative thinking activities [105]. For sports undergraduates, who often face the dual challenges of intensive physical training and theoretical learning, generative AI helps alleviate the cognitive burden by handling energy-consuming foundational tasks such as training data analysis and report drafting (e.g., generating competition analysis reports). Consequently, students are released from the simultaneous cognitive strain of physical training and theoretical analysis, enabling the reallocated cognitive resources to be directly invested in creative processes such as movement innovation and tactical breakthroughs. It is also important to acknowledge that generative AI dependence may not be a homogeneous construct. Different forms of dependence, such as adaptive and reflective reliance and maladaptive or thoughtless overreliance, may lead to different creative outcomes, suggesting more nuanced pathways linking AI use to creativity.

The results further show that generative AI dependence indirectly affects creativity through self-efficacy (β = 0.271, p < 0.001). This finding aligns with the triadic reciprocal framework of “environment–individual–behavior” emphasized in Social Cognitive Theory [58], suggesting that generative AI, as a technologically supported learning environment, can enhance individuals’ internal cognition and thereby stimulate greater behavioral engagement and exploration. The mediating role of self-efficacy is particularly noteworthy. For undergraduates in sport-related disciplines, generative AI dependence enhances their confidence in handling sport-specific tasks and problem-solving, strengthening their ability to demonstrate novel and original thinking in practice.

In addition, the results indicate that the mediating role of flow in the relationship between generative AI dependence and creativity did not reach a significant level (β = 0.019, p = 0.086). This further disrupts the chain mediation path of “generative AI dependence → self-efficacy → flow → creativity”, preventing the formation of an effective transmission mechanism. Although this outcome deviates from the expectations of Flow Theory [106], it can be reasonably explained by the characteristics of the study population. Students in sport-related disciplines emphasize physical practice and immediate feedback, with their flow experiences primarily arising from immersive physical training. In contrast, generative AI dependence functions as a cognitive support tool incompatible with the mechanisms underlying their flow experiences. Moreover, sports undergraduates tend to focus more on the practical outcomes of technology use than on the experiential process, further weakening the mediating role of flow. While generative AI tools significantly enhance students’ self-efficacy in completing tasks, this increase in confidence does not effectively translate into a deeply immersive flow state, impeding the pathway through which self-efficacy might otherwise promote creativity via flow. This phenomenon highlights the distinctive trait of sports undergraduates—“prioritizing physical practice while relying on technological tools primarily for outcome-oriented support”—and its specific influence on psychological transmission mechanisms. Given this pattern, the absence of a significant mediating effect is likely rooted in the task characteristics of this population, indicating that the influence of AI on flow may depend heavily on whether the learning activity is cognitively oriented or physically oriented.

About gender differences, the results show that the positive effects of generative AI dependence on both flow and creativity are more pronounced among male students. However, no significant gender difference was observed in the relationship between generative AI dependence and self-efficacy. In other words, dependence on generative AI tools more strongly enhances training-related flow among male students. This may be attributed to men’s generally greater inclination to use AI for self-improvement [107], which motivates them to explore and integrate generative AI tools more actively in their sports training. At the same time, female students tend to experience flow less frequently than their male counterparts during sports activities [108], and this inherent difference in flow frequency further attenuates the effect of generative AI dependence on women’s flow experiences. Moreover, generative AI dependence significantly boosts creativity among male students. This finding is consistent with both Creativity Investment Theory and Social Role Theory. Creativity Investment Theory states innovation requires “swimming against the tide” by taking risks to achieve breakthroughs [109]. Social Role Theory further emphasizes that society places different expectations on men and women, with men being more strongly encouraged to display initiative, risk-taking, and norm-challenging behaviors [75]. Within the sports context, this can be reasonably explained by the competitive and breakthrough-oriented nature of the field, which makes societal expectations more likely to encourage men to treat generative AI tools as a means of “adventurous breakthroughs” (e.g., devising innovative tactics or training programs), thereby catalyzing creativity. By contrast, women may be more constrained by normative role expectations, leading to a more conventional use of generative AI that diminishes its creative potential. However, the effect of generative AI dependence on enhancing self-efficacy does not differ by gender. This outcome deviates from the predictions of Social Cognitive Theory [110], but it can be reasonably interpreted in the sports context. Progress in sports training is highly measurable; regardless of gender, when students receive personalized training programs from generative AI and observe tangible improvements, the “seeing is believing” mechanism produces a similar confidence-boosting effect, resulting in comparable increases in self-efficacy for both male and female students.

### Theoretical implications

First, this study is the first to introduce generative AI dependence as a key variable into the research field of creativity among sports undergraduates, thereby addressing the lack of theoretical exploration of emerging technologies in the literature. Previous studies have primarily focused on the role of generative AI as a tool or collaborator in influencing creativity [111], while overlooking the unique patterns of dependence it induces and their consequences. By empirically verifying the effect of generative AI dependence on the creativity of sports undergraduates, this study provides a novel perspective for understanding how generative AI becomes deeply embedded in human creative activities. Future research may extend this construct to other athletic populations to further test the cross-group generalizability of the theory.

Second, this study indicate that generative AI dependence not only exerts a direct effect on creativity but also influences it indirectly through the mediating role of self-efficacy, whereas the mediating effect of flow was found to be non-significant. Specifically, this study validates the direct pathway from generative AI dependence to enhanced creativity in the context of sports education, thereby extending existing research that has predominantly focused on business, general education, and the arts. In addition, the identification of self-efficacy as a critical psychological mechanism highlights its central role in the transmission pathways linking technology use and creativity. By contrast, the unexpectedly non-significant mediating role of flow suggests that theoretical frameworks emphasizing flow may not be universally applicable to the sports education context, thereby expanding the theoretical boundary of current explanations. Taken together, these results enrich the understanding of how technology use affects creativity and provide new directions for future research to examine alternative psychological and behavioral mediators.

Third, through multi-group comparisons, this study reveals gender heterogeneity in the effects of generative AI dependence on core psychological constructs of sports undergraduates (flow and creativity), indicating that gender plays a critical moderating role in the relationship between technological dependence, flow, and creativity. Existing research has paid limited attention to the moderating mechanism of gender in the link between technology dependence and flow or creativity, particularly overlooking the interactive effects of gender and technology-use tendencies in the sports domain. By examining gender’s moderating effects through both the affective pathway (flow) and the cognitive pathway (creativity), this study extends the population boundaries of research on technology’s influence on psychological traits and uncovers a multi-pathway pattern of gender moderation. Future studies may further investigate the underlying mechanisms that drive these gender differences.

### Practical implications

This study examined the impact of generative AI dependence on the creativity of sports undergraduates and found that generative AI dependence exerts a significant positive direct effect on creativity and an indirect effect through self-efficacy. At the same time, the mediating role of flow was not significant. In addition, the results revealed that the positive effects of generative AI dependence on flow and creativity were more pronounced among male students. These findings enrich the generative AI use and creativity literature and hold important practical value for policymakers, educational institutions, and sports undergraduates.

In light of the finding that generative AI dependence has a direct positive impact on the creativity of sports undergraduates, policymakers should promote the widespread adoption of AI technologies in the sports domain. In doing so, enhancing students’ creativity should be regarded as a key learning goal in sports education. Specifically, the government could increase investment in AI infrastructure for sports universities, such as equipping institutions with advanced AI-based sports training analysis systems. Additionally, policymakers may introduce initiatives to promote the integration of AI technologies in sports education, encouraging universities to adopt and apply AI tools actively. These policies can help ensure that AI is used not only for performance monitoring but also for facilitating higher-order tactical innovation and movement creativity.

This study demonstrates that generative AI dependence influences the creativity of sports undergraduates indirectly through self-efficacy, suggesting that educational institutions should focus on enhancing students’ self-efficacy in using generative AI tools. Specifically, universities could organize skill competitions on generative AI applications, enabling students to accumulate successful experiences through practice, or showcase case studies of sports innovation achieved with generative AI, thereby strengthening students’ confidence in using such tools effectively. In addition, institutions may incorporate generative AI-assisted training tasks into routine teaching, allowing students to build self-efficacy through task completion and gradually promoting creativity development. Moreover, instructors should explicitly highlight students’ successful applications of AI tools in real sports learning contexts, reinforcing efficacy beliefs and sustaining motivation for creative task engagement.

The results of this study indicate that generative AI dependence indirectly affects creativity through self-efficacy. Thus, creating conditions under which students gain a sense of mastery using generative AI is crucial for turning confidence into creative performance. Based on this finding, instructors in sports education should design AI-supported learning tasks that progressively build students’ self-efficacy before engaging them in creative production. For instance, initial tasks could involve critically evaluating AI-generated training plans or tactical suggestions. Once students have established self-efficacy, instructors can introduce more open-ended, creative tasks such as designing innovative training programs, producing multi-perspective tactical analyses, or creating sport-related digital content with generative AI. This stepwise approach ensures that students internalize adaptive AI use patterns, leverage their self-efficacy, and ultimately enhance their creative performance in sports learning contexts. This scaffolding-based instructional approach is essential for avoiding maladaptive overreliance and fostering productive, creativity-enhancing AI use.

The results of this study show that gender significantly moderates the impact of generative AI dependence on both flow and creativity. Given that the pathways from AI dependence to psychological experience and creativity vary by gender, differentiated instructional strategies are recommended. For male sports undergraduates, it is recommended to directly leverage generative AI tools—such as ChatGPT to design personalized training programs or Midjourney to create visual training materials—to enhance flow experiences, while employing these tools to develop innovative training methods that strengthen creativity. A gradual approach is advisable for female sports undergraduates: starting with basic generative AI applications, such as using generative AI to record training data or generate simple training plans, to build trust and familiarity progressively. Participation in collaborative projects—such as teams using generative AI to analyze competition data and devise strategies—can further enhance their competence in generative AI applications, fostering stronger flow experiences and greater creativity. Additionally, instructors should be attentive to students’ individual comfort levels and technology-related anxiety to ensure that AI-driven creativity promotion is inclusive for all learners.

### Limitations and future research

While this study provides valuable insights into the impact of generative AI dependence on sports undergraduates’ creativity, several limitations warrant attention in future research. First, the cross-sectional and self-reported design restricts causal inference and may introduce common method variance, suggesting that future research should employ longitudinal or experimental designs and adopt procedural or statistical remedies to mitigate bias. In addition, the exclusive use of quantitative methods, while effective for hypothesis testing, limits the ability to capture the nuanced psychological experiences associated with AI dependence; thus, integrating qualitative approaches such as interviews or focus groups could yield richer contextual understanding. The sample’s gender imbalance, regional homogeneity, and reliance on snowball sampling within a single province further constrain external validity, highlighting the need for larger and more diverse samples across multiple regions and institutions, as well as the assessment of measurement invariance across subgroups. Moreover, this study did not distinguish participants by specific sport majors, although differences between team sports (e.g., soccer) and individual disciplines (e.g., Tai Chi) may influence psychological processes and technology-use patterns, warranting more fine-grained subgroup analyses in future work. Finally, because self-efficacy was examined as the sole mediator, future studies should incorporate additional mechanisms—such as psychological safety or regulated learning—and further clarify when generative AI dependence is adaptive or maladaptive, as well as investigate whether task-type characteristics (e.g., cognitive vs. physical tasks) moderate the observed relationships to achieve a more comprehensive understanding of how generative AI shapes creativity.

## Conclusion

This study aimed to examine the impact of generative AI dependence on the creativity of sports undergraduates, with a particular focus on the mediating roles of self-efficacy and flow. Data were collected from 453 undergraduate students majoring in sports in Jiangxi Province, China, and analyzed using structural equation modeling. The results show that generative AI dependence significantly affects creativity, with self-efficacy as a partial mediator in this relationship. Multi-group analysis further revealed that the positive effects of generative AI dependence on flow and creativity are more pronounced among male students. By focusing on the sports education context, this study is the first to demonstrate that generative AI dependence fosters creativity among sports undergraduates through enhancing self-efficacy, and that this effect exhibits significant gender differences. It fills an important gap in the literature on technology dependence in sports education and among students in sport-related disciplines. Nevertheless, limitations such as the gender imbalance and regional homogeneity of the sample and the cross-sectional design may constrain the generalizability and depth of the findings. Future research should address these limitations to enhance this line of inquiry’s comprehensiveness and practical relevance.

## Supporting information

S1 Appendix Items Used in the Questionnaire. This appendix includes all questionnaire items referenced in the manuscript, providing the full set of survey questions used to collect data from sports undergraduates.
(DOCX)

S1 Data Raw data. (XLSX)
