# Supplementary material for: Does generative AI dependence foster creativity? Evidence from sports undergraduates
Source: PLoS One. 2026 Feb 9;21(2):e0341277. doi: 10.1371/journal.pone.0341277 (PMC12885276; doi:10.1371/journal.pone.0341277)
Supplement: S1 Appendix — This appendix includes all questionnaire items referenced in the manuscript, providing the full set of survey questions used to collect data from sports undergraduates. (DOCX) [file pone.0341277.s001.docx]

**S1.** Items Used in the Questionnaire

| **Construct** | **Item** | **Item** | **References** |
| --- | --- | --- | --- |
| Generative AI dependency | **Reflective use** | I designed my own prompts for Generative AI so it could give me the output I needed. | Hou et al. (2025) |
|  |  | I critically evaluate the output generated by AI tools. |  |
|  |  | I revised my prompting questions so Generative AI can give more appropriate outputs. |  |
|  |  | I asked Generative AI to improve my initial draft/solutions for the problem-solving activities. |  |
|  | Cautious use | I spotted errors in the Generative AI’s output. |  |
|  |  | I recognize that the outputs generated by AI tools are not perfect |  |
|  |  | I discarded Generative AI’s output. |  |
|  |  | I finished a subtask in problem-solving activities with no or limited help from Generative AI. |  |
|  | Thoughtless use | I copied the task descriptions or problem statements to Generative AI for help. |  |
|  |  | I copied the Generative AI’s output as part of our solution |  |
|  |  | For each activity I did in this class, I spent most of the time interacting with Generative AI. |  |
|  |  | I directly copy AI-generated content into my solutions without modification |  |
|  | Collaborative use | My peers help me spot error in the Generative AI’s output. |  |
|  |  | My peers help me revise the output produced by generative AI tools |  |
|  |  | I asked my peer(s) questions during the problem-solving activities. |  |
|  |  | My peers answered my questions and supported my understanding. |  |
| Self-efficacy | SE1 | I believe I can fluently use generative AI to complete my required tasks. | Fangzhou et al. (2024) |
|  | SE2 | I believe I can quickly master the operation methods and interaction techniques of generative AI. |  |
|  | SE3 | I believe I can complete required tasks more effectively with the help of generative AI |  |
| Flow | FL1 | When interacting with generative AI, I think about nothing else. | Bakker and van Woerkom (2017) |
|  | FL2 | When interacting with generative AI, I get carried away by the activity. |  |
|  | FL3 | When interacting with generative AI, I forget everything else around me. |  |
|  | FL4 | When interacting with generative AI, I enter a state of complete absorption. |  |
| Creativity | CRE1 | With the help of generative AI, I can propose new ways to achieve my goals | Tan and Ong (2019) |
|  | CRE2 | With the help of generative AI, I can discover new approaches to enhance my capabilities. |  |
|  | CRE3 | With the help of generative AI, I can consistently generate creative ideas. |  |
|  | CRE4 | With the help of generative AI, I can identify new perspectives for solving problems. |  |
|  | CRE5 | With the help of generative AI, I can propose innovative ways to complete my tasks. |  |

*Note: The original English version was translated into Chinese and slightly adapted to fit the context of the study. However, the underlying constructs and meanings of the items remained unchanged.*

Bakker, A. B., & van Woerkom, M. (2017). Flow at Work: a Self-Determination Perspective. *Occupational Health Science*, *1*(1), 47-65. <https://doi.org/10.1007/s41542-017-0003-3>

Fangzhou, J., Lin, C.-H., & Lai, C. (2024). Modeling AI-Assisted Writing: How Self-Regulated Learning Influences Writing Outcomes. *Computers in Human Behavior*, *165*, 108538. <https://doi.org/10.1016/j.chb.2024.108538>

Hou, C., Zhu, G., Sudarshan, V., Lim, F. S., & Ong, Y. S. (2025). Measuring undergraduate students' reliance on Generative AI during problem-solving: Scale development and validation. *Computers & Education*, *234*, 105329. <https://doi.org/10.1016/j.compedu.2025.105329>

Tan, C.-S., & Ong, A. W.-H. (2019). Psychometric Qualities and Measurement Invariance of the Modified Self-Rated Creativity Scale. *The Journal of Creative Behavior*, *53*(4), 593-599. <https://doi.org/https://doi.org/10.1002/jocb.222>
